# Magnified endoscopy with texture and color enhanced imaging with indigo carmine for superficial nonampullary duodenal tumor: a pilot study

**DOI:** 10.1038/s41598-022-14476-4

**Published:** 2022-06-20

**Authors:** Kenichiro Okimoto, Tomoaki Matsumura, Daisuke Maruoka, Akane Kurosugi, Wataru Shiratori, Ariki Nagashima, Tsubasa Ishikawa, Tatsuya Kaneko, Kengo Kanayama, Naoki Akizue, Yuki Ohta, Takashi Taida, Keiko Saito, Jun Kato, Naoya Kato

**Affiliations:** 1 Department of Gastroenterology, Graduate School of Medicine, Chiba University, Inohana 1-8-1, Chiba-City, 260-8670 Japan; 2 Kameido Endoscopy and Gastroenterology Clinic, Tokyo, Japan

**Keywords:** Cancer imaging, Gastrointestinal cancer

## Abstract

This pilot study aimed to investigate the utility of texture and color enhancement imaging (TXI) with magnified endoscopy (ME) for the preoperative diagnosis of superficial nonampullary duodenal epithelial tumors (SNADETs). We prospectively evaluated 12 SNADETs. The visibility for ME-TXI, ME with indigo carmine (ICME)—white-light imaging (WLI), ICME-TXI compared to ME-NBI (narrow-band imaging) was scored (+ 2 to − 2 ME-NBI was set as score 0) by 3 experts. Scores + 2 and + 1 were defined as improved visibility. The intra-observer and interobserver agreement for improved visibility of surface structure (SS) was evaluated. Sensitivity, specificity, and positive predictive value (PPV) for Vienna Classification (VCL) C4/5 associated with the preoperative diagnosis of ICME-TXI were analyzed. The SS visibility score of ICME-TXI was significantly higher than that of ME-NBI, ME-TXI, and ICME-WLI (*P* < 0.001 respectively). The kappa coefficients of reliability for intra-observer and interobserver agreement for the SS visibility improvement with ICME-TXI were 0.96, 1.00, 1.00 and 0.70, 0.96, 0.96 respectively. All endoscopists preferred ICME-TXI for visualizing SS mostly for all lesions. The sensitivity, specificity, and PPV (%) of ICME-TXI for VCL C4/5 were 80, 66.7, and 63.2, respectively. ICME-TXI facilitates the visibility of the SS of SNADETs and may contribute to their preoperative diagnosis.

## Introduction

The incidence of identified superficial nonampullary duodenal epithelial tumors (SNADETs) has gradually increased owing to advancements in endoscopic technology^1^. Pancreaticoduodenectomy (PD) is a standard treatment for duodenal cancers. However, PD is an invasive treatment with a mortality rate ranging from 1 to 4%^2,3^. Therefore, it is very important to accurately diagnose SNADETs to allow the less invasive management such as underwater endoscopic mucosal resection^4–6^ or endoscopic submucosal dissection^7–10^.

The accuracy of preoperative biopsy for SNADETs is unsatisfactory, ranging from 68 to 71.6%^11,12^. In addition, small biopsy bites for SNADETs can cause severe fibrosis, which may become later obstacles for endoscopic resection^11^. Nowadays, there are more studies of the utility of image-enhanced endoscopy (IEE) for SNADETs. Magnified endoscopy (ME) with narrow-band imaging (NBI) for SNADETs is useful^13–15^. Further, the accuracy of diagnosis for the Vienna Classification (VCL) C3, C4 or C3, C4/5^16,17^ has been reported to range from 65.1 and 87%^13–15^, suggesting that endoscopic diagnosis is at least comparable to biopsy.

In 2020, Olympus Medical Systems Corporation (Tokyo, Japan) introduced a new IEE system for texture and color enhancement imaging (TXI). In TXI, an image usually obtained by white-light irradiation is divided into a texture image and a base image. After enhancing the texture and correcting the color tone and brightness, the images are combined^18^. We previously reported the usefulness of TXI for visualizing gastric mucosal atrophy and gastric neoplasms^18^. However, its utility with ME for SNADETs has not yet been clarified.

Thus, this pilot study aimed to investigate the utility of TXI with ME for the preoperative diagnosis of SNADETs.

## Methods

### Study design and participants

This prospective study was conducted at Chiba University Hospital (Japan) between March and July 2021. Patients diagnosed with sporadic SNADETs were prospectively enrolled in this study before diagnostic endoscopy. After endoscopic diagnosis, all patients underwent endoscopic resection or an operation. This study was reviewed and approved by the institutional review board of Chiba University School of Medicine and was registered at the University Hospital Medical Information Network (UMIN000041436). Informed consent was obtained from all participants. All methods were performed in accordance with the relevant guidelines and regulations.

### Instruments

We used the CV-1500 light source equipped with a TXI system and the GIF-XZ1200 and GIF-H290Z endoscopes (Olympus Medical Systems Corporation, Tokyo, Japan). There are two types of TXI: mode 1 and mode 2. In TXI mode 1, the color tone changes are more coordinated than in TXI mode 2. In this study, we used only TXI mode 1. For the structure enhanced mode, “A7” was selected for NBI and (white-light imaging) WLI, while “strong” was selected for TXI^18^.

### Magnified endoscopy

ME with WLI (ME-WLI), NBI (ME-NBI), and ME with TXI (ME-TXI) were performed for each lesion. For ME-WLI and ME-TXI, indigo carmine (IC) dye was also used (ICME-WLI, ICME-TXI, respectively). During the preoperative endoscopic diagnosis of SNADETs, the lesions were assessed for size (mm), location in the duodenum, macroscopic findings, endoscopic diagnosis according to VCL^16,17^, and the deposition of white opaque substance (WOS)^19^. The macroscopic findings were assessed according to the Paris endoscopic classification^20^. VCL endoscopic diagnosis was based on the presence of WOS, size of the lesion, and surface structure (SS) (closed- or open-loop) as described in a previous report^15^. Closed-loop structures were defined as oval-shaped mucosal structures that could be interpreted as connecting the starting point of the mucosa to the endpoint. Open-loop structures were defined as linear mucosal structures^15^. Endoscopic diagnosis with ICME-TXI was performed separated by each endoscopist.

### Evaluation of pathology

All the tissue samples were sent for histological analysis with standard hematoxylin and eosin stain and were evaluated by gastrointestinal pathologists. The pathological diagnosis was according to the VCL, with classification into VCL C3 and C4/5. The details of VCL C4/5 are as follows; high-grade adenoma (C4.1), noninvasive carcinoma (C4.2), suspicious for invasive carcinoma (C4.3), intramucosal carcinoma (C5.1), and submucosal carcinoma (C5.2).

### Outcomes

The primary endpoint of this study was the visibility of the SS for SNADETs with ICME-TXI. We also assessed the visibility of the SNADETs’ blood vessels. The visibility for ME-TXI, ICME-WLI, ICME-TXI compared to ME-NBI (ME-NBI was set as score 0) was scored by three experts (an endoscopist with > 5 years of IEE experience) in five stages, a modification of previous reports^21–23^. The details of the score are as follows, + 2 (improved visibility remarkably), + 1 (improved visibility), 0 (unchanged visibility), − 1 (worsened visibility), − 2 (worsened visibility remarkably). Scores + 2 and + 1 were defined as improved visibility. The intra-observer and interobserver agreements for the improved visibility of SS were evaluated. For intra-observer agreement, the first and second evaluations were spaced 6 months apart. In addition, the favored modality by the examiners for the evaluation of the SS and blood vessels was assessed. The sensitivity, specificity, and positive predictive value (PPV) for VCL C4/5 associated with the preoperative diagnosis of ICME-TXI and preoperative biopsy were also investigated.

### Sample size calculation

There were no data regarding ICME-TXI for SNADETs. As this was a pilot study, 12 lesions were enrolled based on criteria from a previous report^24^.

### Statistical analysis

Baseline data were presented as mean ± standard deviation (SD). The differences in visibility scores were analyzed using Friedman's test with Bonferroni correction. Intra-observer and interobserver agreement for the visibility improvement was assessed with Cohen’s Kappa analysis. The kappa coefficient of reliability was classified as follows: 0.0–0.2 (slight agreement), 0.21–0.40 (fair agreement), 0.41–0.60, (moderate agreement), 0.61–0.80 (substantial agreement), and 0.81–1.0 (almost perfect or perfect agreement). All statistical analyses were performed using the Statistical Package for the Social Sciences software version 26 (SPSS Inc., Chicago, IL, USA). *P* values < 0.05 were considered statistically significant.

## Results

### Patients and lesions

**Figure 1:**
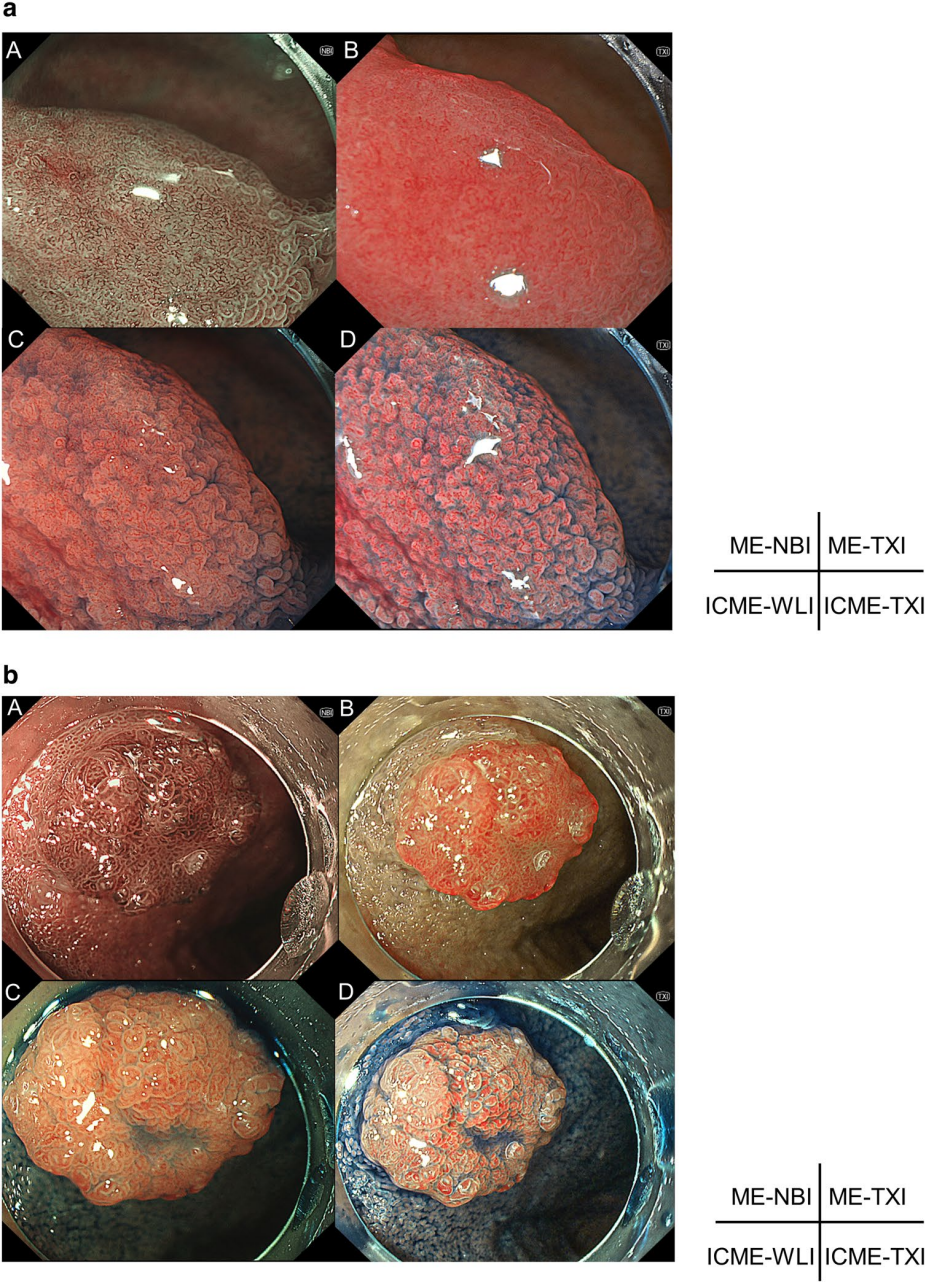
Representative cases of SNADETs for each modality. SNADET, Superficial nonampullary duodenal epithelial tumors; ME-WLI, magnified endoscopy with white-light imaging, *ME-NBI* magnified endoscopy with narrow-band imaging, *ME-TXI* magnified endoscopy with texture and color enhancement imaging, *ICME-WLI* indigo carmine dye with ME-WLI, *ICME-TXI* indigocarmine dye with ME-TXI, *VCL* Vienna classification, *IMC* intramucosal carcinoma. (**a**) 13 mm, IIa, pathological diagnosis of VCL C3. A. ME-NBI. B. ME-TXI. C. ICME-WLI. D. ICME-TXI. Open-loop was observed. (**b**) 10 mm, IIa, pathological diagnosis of VCL C4/5 (IMC). A. ME-NBI. B. ME-TXI. C. ICME-WLI. D. ICME-TXI. Closed-loop was observed.

In total, 11 patients and 12 lesions were evaluated. The characteristics of the patients and lesions are shown in Table 1. The median tumor size was 10 mm. The macroscopic findings were IIa dominant. Seven lesions (58.3%) were VCL C3, and 5 lesions (41.7%) were VCL C4/5. WOS was identified in 8 lesions (66.7%). Representative cases of endoscopic observation in each modality for SNADETs are shown in Fig. 1a, b.

**Table 1:** Characteristics of the participants and lesions.

	All patients
	(n = 11)
Age (years), median (range)	69 (37–80)
Male/female	5/6

	All lesions
	(n = 12)
Size (mm), median (range)	10 (3–100)
**Location of the lesions, n (%)**	
2nd (oral side of the major papilla)	8 (66.7)
2nd (anal side of the major papilla)	4 (33.3)
**Macroscopic findings, n (%)**	
Is	2 (16.7)
IIa	9 (75.0)
IIc	1 (8.3)
**Histopathological diagnosis, n (%)**	
VCL C3	7 (58.3)
VCL C4/5	5 (41.7)
Presence of WOS, n (%)	8 (66.7)
**Resection, n (%)**	
CSP	4 (33.3)
UEMR	6 (50.0)
Surgical operation	2 (16.7)

*VCL* Vienna classification, *CSP* cold snare polypectomy, *UEMR* underwater endoscopic mucosal resection.

### Visibility score

**Figure 2:**
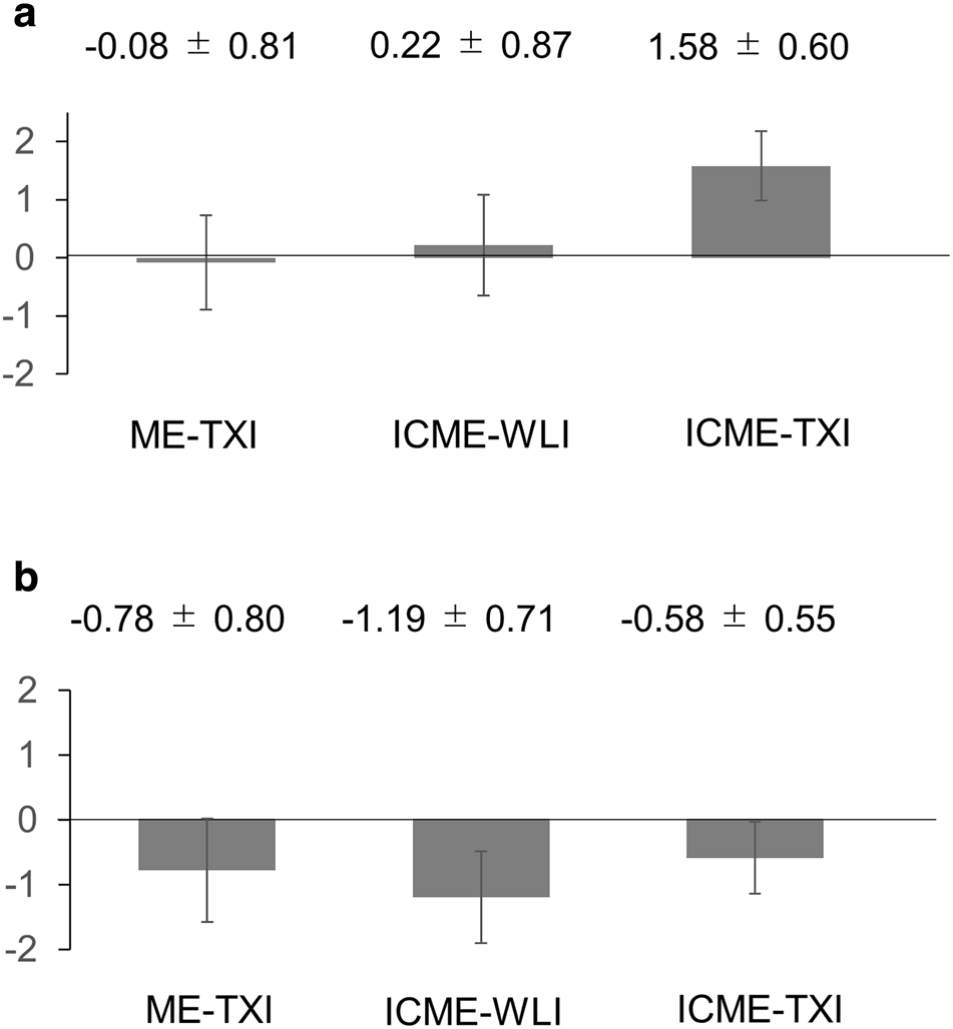
The mean visibility scores (described mean ± SD) for each modality. The mean visibility score of ME-NBI was set as score 0. *SD* standard deviation, *ME-WLI* magnified endoscopy with white-light imaging, *ME-NBI* magnified endoscopy with narrow-band imaging, *ME-TXI* magnified endoscopy with texture and color enhancement imaging, *ICME-WLI* indigo carmine dye with ME-WLI, *ICME-TXI* indigocarmine dye with ME-TXI. (**a**) Mean visibility score for surface structure. Mean visibility scores for ME-TXI, ICME-WLI, ICME-TXI were − 0.08 ± 0.81, 0.22 ± 0.87, 1.58 ± 0.60, respectively. (**b**) Mean visibility score for blood vessels. Mean visibility score for ME-TXI, ICME-WLI, ICME-TXI were − 0.78 ± 0.80, − 1.19 ± 0.71, − 0.58 ± 0.55, respectively.

The mean visibility scores (± SD) in each modality are shown in Fig. 2a, b. As described previously, the mean visibility score of ME-NBI was set as 0. For visibility of SS, the mean visibility scores for ME-TXI, ICME-WLI, ICME-TXI were − 0.08 ± 0.81, 0.22 ± 0.87, 1.58 ± 0.60, respectively. For the visibility score of blood vessels, the mean visibility score for ME-TXI, ICME-WLI, ICME-TXI were − 0.78 ± 0.80, − 1.19 ± 0.71, − 0.58 ± 0.55, respectively. The *P* value of the visibility scores of SNADETs in ME-TXI, ICME-WLI, and ICME-TXI compared to ME-NBI are shown in Table 2a, b. In summary, the visibility score of SS in ICME-TXI was significantly higher than those of ME-NBI, ME-TXI, and ICME-WLI (*P* < 0.001, respectively). The visibility scores of blood vessels in ME-TXI, ICME-WLI, ICME-TXI were significantly lower than ME-NBI (*P* = 0.001, *P* < 0.001, *P* = 0.028, respectively). In addition, the visibility score of blood vessels in ICME-TXI was significantly higher than ICME-WLI (*P* = 0.003).

**Table 2:** *P* value of visibility scores of SNADETs in ME-TXI, ICME-WLI, and ICME-TXI compared to ME-NBI.

*P* value					
ME-NBI vs ME-TXI	ME-NBI vs ICME-WLI	ME-NBI vs ICME-TXI	ME-TXI vs ICME-WLI	ME-TXI vs ICME-TXI	ICME-WLI vs ICME-TXI
(a) *P* value of visibility scores for surface structure					
1.000	1.000	< 0.001	1.000	< 0.001	< 0.001
(b) *P* value of visibility scores for blood vessels					
0.001	< 0.001	0.028	0.082	1.000	0.003

The ME-NBI was set as score 0.

*P* value was calculated using Friedman’s test with Bonferroni correction.

*SNADETs* superficial nonampullary duodenal epithelial tumors, *ME-WLI* magnified endoscopy with white-light imaging, *ME-NBI* magnified endoscopy with narrow-band imaging, *ME-TXI* magnified endoscopy with texture and color enhancement imaging, *ICME-WLI* indigo carmine dye with ME-WLI, *ICME-TXI*, indigocarmine dye with ME-TXI.

### Preference of modality for surface structure and blood vessels

For visualizing SS, all endoscopists preferred ICME-TXI mostly for all lesions. On the other hand, for visualizing blood vessels, all endoscopists preferred ME-NBI mostly for all lesions.

### Intra-observer and interobserver agreement for the improvement of visibility for surface structure with ICME-TXI

The kappa coefficients of reliability for intra-observer agreements for the visibility improvement for SS with ICME-TXI were 0.96, 1.00, and 1.00. While those of interobserver agreement for the visibility improvement for SS with ICME-TXI were 0.70, 0.96, and 0.96. Intra-observer agreements for SNADETs were “almost perfect or perfect agreement”. Interobserver agreements for SNADETs were “substantial agreement” or “almost perfect or perfect agreement.”

### Sensitivity and PPV for VCL C4/5 in association with preoperative diagnosis of ICME-TXI

The sensitivity, specificity, and PPV (%) for VCL C4/5 associated with a preoperative diagnosis of ICME-TXI (calculated for 36 lesions, 12 for each endoscopist) were 80, 66.7, and 63.2, respectively. Preoperative biopsy was performed in 9 of 12 lesions. The sensitivity, specificity, and PPV (%) for VCL C4/5 in preoperative biopsy were 40.0, 75.0, and 66.7, respectively. The sensitivity of preoperative diagnosis with ICME-TXI for SNADETs tended to be higher than with preoperative biopsy.

## Discussion

This study is the first to report the usefulness of ME-TXI, including ICME-TXI. Conventionally, NBI was the main modality for ME^13–15^.

The visibility of the SS with ICME-WLI and ME-TXI did not differ from that of ME-NBI. But with ICME-TXI, the SS were more visible than with ME-NBI. The visibility of the SS did not improve with IC or texture enhancement of TXI alone. It is thought that accumulated IC in the concave parts of the SNADETs’ surface made the ductal structure outline stand out due to the color emphasis. This made the SS easy to recognize. The kappa coefficients of reliability for interobserver agreement for improving visibility for SS of ICME-TXI and the percentage for endoscopists’ preference for ICME-TXI regarding the recognition of the SS were high. It seems that the reproducibility is high. Clinical effects of the results in this study were considered as follows. First, better visibility of ICME-TXI for SS might allow easier preoperative diagnosis of SNADETs even for non-expert. Second, a new diagnostic system might be constructed on the basis of clearly identified SS in the future. Akazawa et al. reported that SNADETs showed distinct endoscopic features according to the mucin phenotype^25^. For instance, the gastric phenotype had dense pattern dilatation of the intervening part with ME-NBI^25^. Although this study did not analyze the mucin phenotype, it could be possible to distinguish mucus phenotype preoperatively by observing the SS with ICME-TXI.

In the previous report of the ME-NBI diagnosis of SNADETs referred to for the diagnosis in this study, the sensitivity, specificity for VCL C4/5 in association with the preoperative diagnosis were 90.5% and 52.4% respectively^15^, which were comparable with our results. The sensitivity of preoperative diagnosis for ICME-TXI tended to be superior to that of biopsy in this study. ICME-TXI can recognize the SS with ease, and preoperative diagnosis may be made more efficiently.

NBI has an absorption range wavelength that matches the blood vessels. Capillaries in the superficial mucosal layer are emphasized by the 415 nm light and are displayed in brown, whereas deeper mucosal and submucosal vessels are visualized by the 540 nm light and are displayed in cyan^26^. Therefore, ME-NBI was still superior in vascular visibility to any other modality in this study. Interestingly, visibility scores for blood vessels of ME-TXI tended to be higher than ICME-WLI. In addition, those of ICME-TXI were significantly higher than with ICME-WLI. The outline of the blood vessel is emphasized by the texture enhancement of TXI and contrast with IC as well, so the blood vessels were easier to identify than with ICME-WLI. The diagnostic method used in this study does not include evaluating blood vessels, but TXI may be useful for diagnosis including assessing the vascular structure.

This study had several limitations. First, it was a pilot study with a sample of only 12. Therefore, the results of this study only suggested the potential of ICME-TXI in the preoperative diagnosis of SNADETs. The results of visibility score of surface structures for ICME-TXI and visibility score of blood vessels for ME-NBI that show significant differences compared to other modalities clearly were considered to be credible. On the contrary, visibility score for blood vessels of ICME-TXI was need to be analyzed with larger samples. Second, the diagnostic method referred to in this study was originally for ME-NBI. A large prospective study using a specific method for TXI for the preoperative diagnosis of SNADETs is crucial for assessing the true value of ICME-TXI.

In conclusion, ICME-TXI facilitates the visibility of the SS of SNADETs and may contribute to the preoperative diagnosis of SNADETs.

## Data Availability

The datasets generated and/or analyzed during the current study are not publicly available due to the protection of personal information.
